# 4085 TL1 Team Approach to Predicting Short-term and Long-term Effects of Spinal Cord Stimulation

**DOI:** 10.1017/cts.2020.362

**Published:** 2020-07-29

**Authors:** Kyle See, Rachel Louise Mahealani Judy, Stephen Coombes, Ruogu Fang

**Affiliations:** 1 University Of Florida Clinical and Translational Science Institute; 2 University of Florida

## Abstract

OBJECTIVES/GOALS: Spinal cord stimulation (SCS) is an intervention for patients with chronic back pain. Technological advances have led to renewed optimism in the field, but mechanisms of action in the brain remain poorly understood. We hypothesize that SCS outcomes will be associated with changes in neural oscillations. METHODS/STUDY POPULATION: The goal of our team project is to test patients who receive SCS at 3 times points: baseline, at day 7 during the trial period, and day 180 after a permanent system has been implanted. At each time point participants will complete 10 minutes of eyes closed, resting electroencephalography (EEG). EEG will be collected with the ActiveTwo system, a 128-electrode cap, and a 256 channel AD box from BioSemi. Traditional machine learning methods such as support vector machine and more complex models including deep learning will be used to generate interpretable features within resting EEG signals. RESULTS/ANTICIPATED RESULTS: Through machine learning, we anticipate that SCS will have a significant effect on resting alpha and beta power in sensorimotor cortex. DISCUSSION/SIGNIFICANCE OF IMPACT: This collaborative project will further the application of machine learning in cognitive neuroscience and allow us to better understand how therapies for chronic pain alter resting brain activity.

